# The Invasion of Alien Populations of *Solanum elaeagnifolium* in Two Mediterranean Habitats Modifies the Soil Communities in Different Ways

**DOI:** 10.3390/plants12112193

**Published:** 2023-05-31

**Authors:** Maria Karmezi, Nikos Krigas, Efimia M. Papatheodorou, Maria D. Argyropoulou

**Affiliations:** 1 Department of Zoology, School of Biology, Faculty of Sciences, Aristotle University, 54124 Thessaloniki, Greece; 2 Institute of Plant Breeding and Genetic Resources, Hellenic Agricultural Organization Demeter, 57001 Thessaloniki, Greece; nikoskrigas@gmail.com; 3 Department of Ecology, School of Biology, Faculty of Sciences, Aristotle University, 54124 Thessaloniki, Greece; papatheo@bio.auth.gr

**Keywords:** diversity profiles, ecosystem disturbance, invasiveness, kermes oak shrublands, microbial PLFA, nematode feeding groups, nematode indices, non-native plants, soil food web

## Abstract

We aimed to explore how the invasion of the alien plant *Solanum elaeagnifolium* affects soil microbial and nematode communities in Mediterranean pines (*Pinus brutia*) and maquis (*Quercus coccifera*). In each habitat, we studied soil communities from the undisturbed core of both formations and from their disturbed peripheral areas that were either invaded or not by *S. elaeagnifolium*. Most studied variables were affected by habitat type, while the effect of *S. elaeagnifolium* was different in each habitat. Compared to maquis, the soil in pines had higher silt content and lower sand content and higher water content and organic content, supporting a much larger microbial biomass (PLFA) and an abundance of microbivorous nematodes. The invasion of *S. elaeagnifolium* in pines had a negative effect on organic content and microbial biomass, which was reflected in most bacterivorous and fungivorous nematode genera. Herbivores were not affected. In contrast, in maquis, organic content and microbial biomass responded positively to invasion, raising the few genera of enrichment opportunists and the Enrichment Index. Most microbivores were not affected, while herbivores, mostly *Paratylenchus,* increased. The plants colonizing the peripheral areas in maquis probably offered a qualitative food source to microbes and root herbivores, which in pines was not sufficient to affect the much larger microbial biomass.

## 1. Introduction

Invasive plants are known to modify plant species communities [1,2] and alter ecosystem productivity [3,4] and abiotic soil properties such as soil nutrients dynamics or soil texture [5,6,7,8] and communities of soil organisms [9,10]. Understanding the interactions and feedbacks between invasive plants and soil biota is a crucial step for the successful management of alien plants in their non-native range. Indeed, during the last two decades, there has been a growing research interest in the effects of plant invasions on components of the soil food web, especially soil microbiomes and nematodes, which are the main counterparts of significant soil processes, e.g., decomposition of organic residues, nitrogen mineralization and cycling, and formation of humic substances, among others [11,12,13,14].

Invasive plants are assumed to experience more positive feedback from soil biota than their native antagonists, possibly due to mutualistic symbionts and/or lower root pathogen pressure [9,15,16,17]. Plant invasions interact with other human-induced changes in the environment [18] and are often facilitated by anthropogenic disturbance [19,20]. However, the role of plant-soil feedback in invasion success may not be straightforward [21,22]. The effects of plant invasions on soil communities may vary according to plant species, locations, year and season of sampling [23,24,25,26]. Most importantly, plant invasions depend on the characteristics of the invaded sites. There are numerous examples where the responses of soil communities varied according to the type of invaded ecosystem. Previous studies [5] have found that *Bromus tectorum* L. invasion in two different grassland associations led to opposite responses from soil bacteria, fungi and nematodes. The invasion of *Heracleum sosnowskyi* Manden. in different habitats is known to cause varied responses in certain nematode trophic groups [27], with the responses of microbial properties being more affected by ecosystem type [28]. Other studies have shown that *Falopia japonica* (Houtt.) Ronse Decr. invasion affects the enzymatic activities differently in dissimilar ecosystems without the overall negative effects of invasion being obscured by the variable response of nematode genera [29]. The invasion by *Solidago gigantea* Aiton in different ecosystems is known to affect either positively or negatively several nematode community indicators, depending on the type of the invaded ecosystem [30]. Other studies have shown that the soil microbial community of coniferous forests is more sensitive than that of deciduous ones to the invasion of *Impatiens glandulifera* Royle [31].

*Solanum elaeagnifolium* Cav. (Solanaceae) is a problematic weed both in its homeland (America), where it is native, as well as in its alien range [32,33]. It invades a vast variety of habitats and has negative effects on local plant communities [34]. It is encountered mainly on disturbed lands [35,36], possibly facilitated by disturbance regimes [37,38]. In Greece, *S. elaeagnifolium* is among the most prominent invasive alien plant species [38,39] and its range has increased by 1750% during the last decades [38]. Research concerning *S. elaeagnifolium* to date has mainly focused on its invasion pattern [38,40], morphological variation, evolution, and genetic differentiation as factors contributing to its adaptability and invasive potential [41,42,43], competitive ability against crops [44,45,46] and ability to interfere with ecosystem services such as pollination [47,48] and crop production as a host of plant pathogens and pests [49,50]. Studies on the chemical nature of *S. elaeagnifolium* have revealed that its secondary metabolites have an adverse effect against plant parasitic nematodes such as *Heterodera zeae* Koshy, Swarup and Sethi and *Meloidogyne incognita* (Kofold & White) Chitwood [51,52] and may reduce the total counts of rhizosphere bacteria and fungi [53]. The only study regarding the effect of *S. elaeagnifolium* invasion on soil nematodes was conducted recently by Karmezi et al. [54], which found a reduction of nematode diversity and changes in the trophic structure of the soil nematode community during the naturalization process of this alien plant. Therefore, the interactions and feedbacks between different soil biota and this noxious weed remain largely unknown.

The aim of this study was to examine and compare the effects of *S. elaeagnifolium* invasion on the soil communities of two different habitats that are typical of the Mediterranean zone, namely the maquis and more specifically the kermes oak (*Quercus coccifera* L.) shrublands and the thermophilous pine forests (*Pinus brutia* Ten.). Both habitats are lowland vegetation formations that are often subjected to anthropogenic disturbances and invasions by alien plants. More specifically, human activities such as road networks, proximity to residential areas and agricultural activities, e.g., livestock grazing, caused the degradation of the peripheral areas of the studied formations, facilitating the invasion of *S. elaeagnifolium* [37,38,55]. The effects of invasion in this study were quantified by estimating soil pH, water content, organic carbon (C_org_) and organic nitrogen (N_org_), biomass of the microbial groups through phospholipid fatty acid analysis (PLFA), abundance and diversity of soil nematodes, as well as by analyzing the trophic and functional structure of the nematode community and its composition in terms of nematode genera. We hypothesized that the invasion of *S. elaeagnifolium* would alter the soil communities of the two habitats in different ways. The results of the investigation herein aim to offer a better understanding of how *S. elaeagnifolium* affects soil dynamics in different ecosystems and will add knowledge regarding the potential underlying mechanisms of its invasiveness.

## 2. Results

All sites had an acidic pH regardless of the habitat type or the disturbance regime and were characterized by low water availability, very low levels of organic N, and in most cases, low levels of organic C (Table 1). Although the clay, silt and sand percentages differed among sites either due to habitat type and/or disturbance regime, the soil texture was sandy loam in all cases. In Table 1, the effects of habitat type and disturbance regime on soil properties are indicated as generated by PERMANOVA. The results of pairwise comparisons among the disturbance regimes within each habitat type are also provided when significant. The water content was affected only by habitat type, exhibiting lower values in *Quercus* sites (kermes oak shrubland) in comparison to the *Pinus* sites (pine forest). C_org_ and N_org_ were also lower at *Quercus* sites. Among the *Pinus* sites, the lower C_org_ values were recorded in the peripheral sites that were invaded by *S. ealeagnifolium* (Pinv). The same holds for N_org_ values, although in the latter case the differences were not significant. On the contrary, among the *Quercus* sites, the invaded peripheral areas (Qinv) were the ones with the higher C_org_ and N_org_ values, although the differences were significant only in the case of N_org_. Both Qinv and Pinv sites displayed the lowest C_org_/N_org_ ratio.

**Figure 1:**
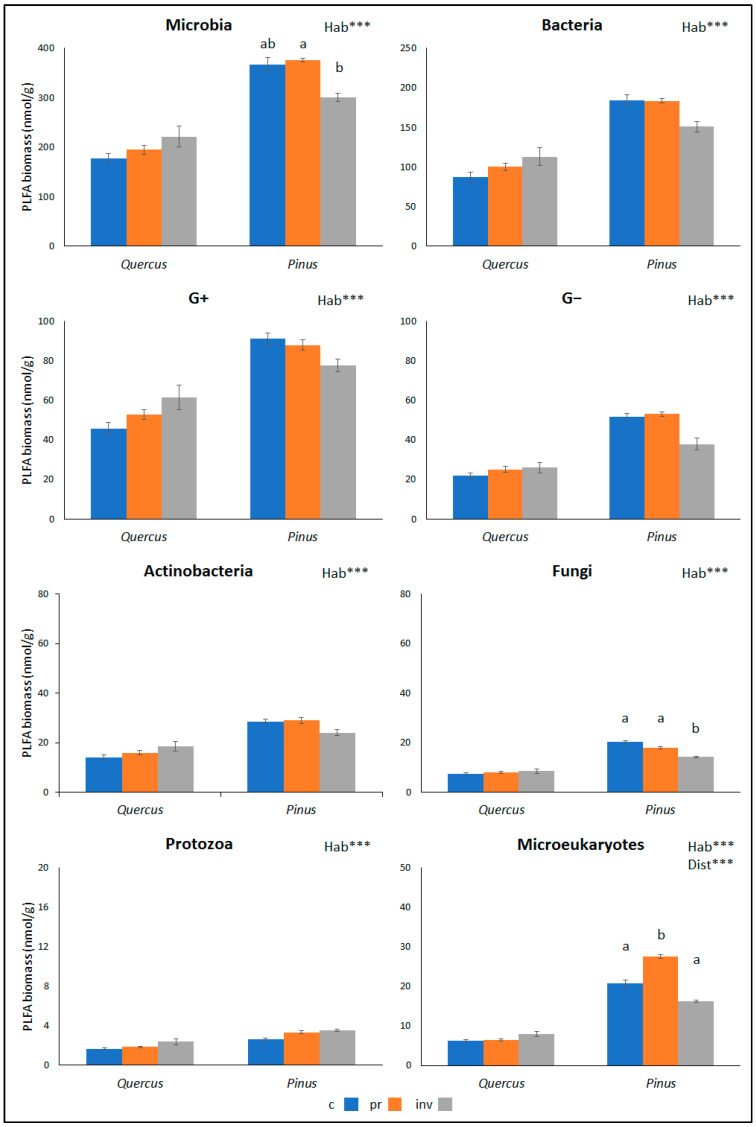
Mean biomass (± SE) of microbial groups in two different habitat types (*Quercus*: *Quercus coccifera* shrublands, *Pinus*: *Pinus brutia* forests) and three different disturbance regimes (c: core, pr: periphery not invaded, inv: periphery invaded by *Solanum elaeagnifolium*). PERMANOVA results for the effects of “habitat” (Hab) and “disturbance” (Dist) are shown (“Dist” nested within “Hab”) (*** *p* < 0.001). Different letters a, b indicate significant differences revealed by pair-wise comparisons between disturbance regimes within each habitat type. For all cases *n* = 4. Raw data are presented in Supplementary Material Table S2.

A total of 35 microbial PLFA biomarkers were extracted from soil samples (Table S1). The effects of habitat type and disturbance regime on the biomasses of the most abundant microbial groups are indicated in Figure 1. In general, the most significant factor influencing the microbial community structure was the type of habitat. The biomasses of all separate PLFA groups as well as the total microbial biomass were smaller in *Quercus* shrublands compared to those in *Pinus* forests. Regarding the disturbance regime, among the *Pinus* sites, the peripheral sites that were invaded by *S. ealeagnifolium* (Pinv) were the ones with the lowest biomass of almost all microbial groups. In the cases of total microbial biomass, fungi and microeukaryotes, this trend was statistically significant. On the contrary, among the *Quercus* shrublands, although the differences due to disturbance regime were not significant, the invaded peripheral sites (Qinv) were the ones with the highest biomass of all microbial groups. Regarding the non-invaded peripheral sites of both habitats (Pinv and Qinv), the biomass of most microbial groups displayed intermediate values.

**Figure 2:**
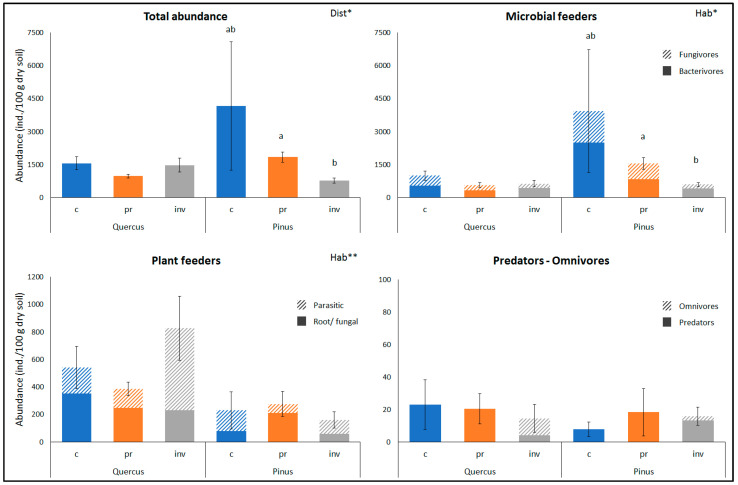
Abundance of nematode trophic groups (mean ± SE) in two different habitat types (*Quercus*: *Quercus coccifera* shrublands, *Pinus*: *Pinus brutia* forests) and three different disturbance regimes (c: core, pr: periphery not invaded, inv: periphery invaded by *Solanum elaeagnifolium*). PERMANOVA results for the effects of “habitat” (Hab) and “disturbance” (Dist) are shown (“Dist” nested within “Hab”) (* *p* < 0.05, ** *p* < 0.01). Different letters a, b indicate significant differences revealed by pair-wise comparisons between disturbance regimes within each habitat type. For all cases *n* = 4. Raw data are presented in Supplementary Material Table S3.

The total nematode abundance and the abundances of nematode trophic groups in the two habitat types and the three disturbance regimes are shown in Figure 2, while the mean values of nematode functional indices are given in Table 2. In general, bacterivores were the most dominant trophic group (more than 30%), followed by the two plant-feeding groups (parasitic and root/fungal feeders) and fungivores. Predatory and omnivorous nematodes had a very low contribution at all sites (around 1%). Omnivores were present only at the sites where *S. elaeagnifolium* had invaded (Qinv and Pinv). The habitat type significantly affected the abundance of the two microbial and the two plant-feeding groups, while the disturbance regime significantly affected the total nematode abundance and that of microbial feeders within the *Pinus* sites, as well as the Enrichment Index in both habitat types. More specifically, the abundance of bacterivores and fungivores was higher at the *Pinus* sites. The inverse holds for herbivores, which were more abundant in the *Quercus* sites (Figure 2). Regarding the effect of the disturbance regime, among the *Pinus* sites, the abundance of microbivores as well as the total nematode abundance and the EI values were lowest in the invaded sites (Pinv) (Figure 2, Table 2). On the other hand, among the *Quercus* sites, the EI values were highest in the invaded sites (Qinv). In most cases, the disturbance effect was intermediate in the peripheral sites that were not invaded by *S. elaeagnifolium*. At this point, we should note that within the *Quercus* sites, the changes in the Channel Index followed the opposite pattern compared to that of the EI, being highest at Qc, lower at Qpr and lowest at Qinv; however, these changes were not statistically significant. The CI values were very high in all studied sites, ranging from 74 to 84, except Qinv (<50).

**Figure 3:**
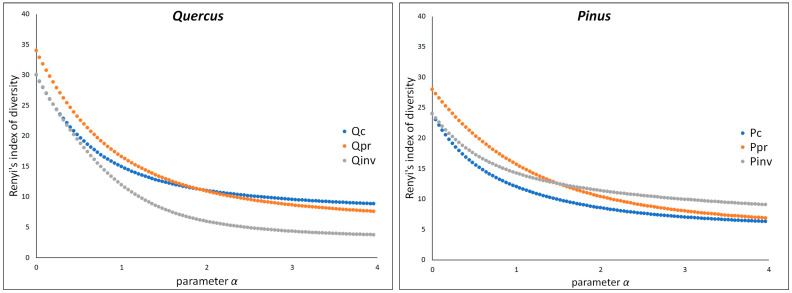
Diversity profiles in two different habitat types (*Quercus*: kermes oak shrublands, *Pinus*: pine forests) and three different disturbance regimes (c: core, pr: periphery not invaded, inv: periphery invaded by *Solanum elaeagnifolium*).

The diversity profiles of nematode communities at all sites are presented in Figure 3. Among the *Quercus* shrublands, the lowest diversity was recorded in the invaded site (Qinv), while the less diverse community among the *Pinus* sites was the one in the undisturbed core of the forest (Pc). In both habitat types, the difference between the core and the peripheral invaded sites (Qc vs. Qinv and Pc vs. Pinv) was not due to the number of genera, as indicated by Renyi’s index at *α* = 0, but due to changes in dominance patterns of abundant genera. In both habitat types, the highest numbers of nematode genera were recorded in the peripheral sites that were not invaded by *S. elaeagnifolium* (Qpr and Ppr).

In Table 3, we provide the PERMANOVA results for the multivariate data sets, namely the whole ensemble of nematode genera and the genera ensemble of each trophic group. For the total nematode community, PERMANOVA revealed significant differences both due to habitat type and disturbance regime. The same holds for the structure of the rest of the trophic groups, except those of predators and omnivores. Pair-wise comparisons within each habitat type revealed that distinct communities were formed in the invaded sites of Qinv and Pinv. The differences between the *Pinus* sites were reflected in the structure of the microbial feeding assemblages, i.e., bacterivores and fungivores, while the differences between the *Quercus* sites were reflected in the structure of the plant feeding groups. We should note that in the case of the plant parasite assemblage, the difference between Qpr and Qinv hardly failed to be significant (*p* = 0.06), and therefore it is not depicted in Table 3. For the same reason, we did not include the difference (*p* = 0.05) in the total nematode community between Qc and Qinv.

For a more detailed description of the nematode communities and to elucidate their above-mentioned differences regarding diversity and genera composition, the mean abundance and percent participation of all nematode genera in the community of each study site are presented in Table 4. A total of 47 nematode genera were recorded across all sites. The bacterivore trophic group was the richest one with 18 nematode genera, followed by that of plant parasites with 11 genera. As mentioned previously, in both habitat types, the highest numbers of nematode genera were recorded in the peripheral sites that were not invaded by *S. elaeagnifolium*: 34 genera at Qpr and 28 genera at Ppr sites. Communities with strong dominance patterns were those at Qinv, which was over-dominated by the phytoparasite *Paratylenchus,* accounting for 37% of the total community, and at “Pc”, where *Acrobeles* and *Ditylenchus* accounted together for more than 40% of the total.

## 3. Discussion

The objective of this study was to explore how the invasion of *S. elaeagnifolium* affects soil communities (nematode and microbial) in two different habitat types that are very characteristic of the Mediterranean region, namely in kermes oak shrublands (*Quercus coccifera*) and pine forests (*Pinus brutia*). In each habitat, we studied soil communities from the relatively undisturbed core of either *Quercus* or *Pinus* formations (Qc, Pc), from the disturbed peripheral areas that have not been invaded yet by *S. elaeagnifolium* (Qpr and Ppr), as well as from the degraded peripheral areas that have been invaded by *S. elaeagnifolium* (Qinv and Pinv). Thus, the studied soil communities derived from sites that differed due to habitat type (Q, P) and disturbance regime (c, pr, inv).

The soil texture at all sites was sandy loam. However, the *Pinus* sites had a higher silt content and a lower sand content compared to the *Quercus* sites, leading to a higher water content. C_org_ and N_org_ were also higher in pine formations. These differences may be important when trying to assess the effects of invasions since the impacts of plant invasions on topsoil chemical properties and soil nutrient pools have been found to be strongly correlated to the initial soil conditions [56]. Previous studies [57] have reported cases where the invasion of the same plant species had different effects on the soil pools of C and N at different sites. Indeed, in this study, we found completely opposite effects regarding the invasion of *S. elaeagnifolium* on C_org_ and N_org_ when comparing the *Pinus* and *Quercus* formations. Although the differences in C_org_ and N_org_ concentrations among sites within the same habitat type were not always significant, a certain pattern of changes was clearly discernible; among the *Pinus* sites, the lowest C_org_ and N_org_ values were recorded in the invaded ones (Pinv), while among the *Quercus* sites, the invaded ones (Qinv) were those with the highest values of C_org_ and N_org_. Our results are in accordance with Dassonville et al. [56], who pointed out that the positive impact of plant invasions, such as higher nutrient concentrations, occurs more often in nutrient-poor sites, while the opposite holds sites with richer soils. Despite the rise and decline of C_org_ and N_org_ in the invaded sites of the “poor” *Quercus* and the “rich” *Pinus* sites, respectively, the C/N values were lowest in the invaded sites of both habitats (Qinv, Pinv), although differences were significant only in the case of Qinv. These findings indicate a rapid mineralization and release of N available for plant uptake [58], which further support the assumption that invasive plants facilitate their own growth by maintaining fast nutrient cycles [59].

As regards the effects of alien plants on soil microbial properties, positive, negative and neutral effects are generally possible, depending on the soil’s initial nutrient status [28]. The changes in microbial biomass among the study sites herein exhibited the same pattern as those in C_org_ and N_org_. The biomass of all microbial groups was significantly higher under pines. Regarding the differences between sites within each habitat type, they were significant only in the case of fungi and only in pines. A higher reduction in total counts of soil fungi than in those of bacteria due to invasion by *S. elaeagnifolium* was also found by Balah et al. [53]. Regarding the rest of the microbial groups in our sites, although the differences due to disturbance regime were not significant, the pattern of changes was once again discernible and reflected that of soil properties, i.e., reduction of microbial biomass at Pinv and increase at Qinv. Many studies have offered evidence of a positive correlation between organic content and microbes, since the former provides food to the latter and controls the development of microbial communities [60]. Moreover, soil bacteria are positively correlated with the nitrogen content of the litter [61]. Other studies [31] indicate that one of the worst alien plants in Europe, namely *I. glandulifera*, can modify soil fungal and bacterial communities via the alteration of soil properties and through the release of allelopathic compounds into the soil. *S. eleagnifolium* is also known for its allelopathic constituents, such as alkaloids [32,62], flavonoids [63,64,65] and terpenes [66]. Moreover, it is known to have antibacterial and antifungal properties [53,67,68]. While this may explain the reduction of microbial biomass at the invaded pine sites herein (Pinv), it contradicts our findings about the microbial increase at the *Quercus* invaded sites (Qinv). Thus, the changes in microbial biomass are more likely to be related to those in soil properties. Changes in above-ground vegetation from the core to the periphery of the *Pinus* and *Quercus* formations might offer an additional explanation. Disturbance has caused the creation of canopy gaps and open spaces in the study sites, allowing the colonization of the understory by many herbaceous plants (Supplementary Material Table S4). These plants have softer tissues that are more easily degraded compared to the more recalcitrant *Pinus* or *Quercus* litter and offer a more labile food source to soil microflora. Therefore, an enrichment of soil and an increase in soil microbes from the core to the peripheral sites, as recorded in the *Quercus* formations, would be expected. But why doesn’t this apply to pines? The answer might lie in the soil conditions that support a much larger microbial biomass in the *Pinus* habitats. Our point is that the microbial enhancement due to colonizing plants that might have been important in Kermes oak shrublands was not equally important in pine forests, where only the invasion of *S. elaeagnifolium* imposed significant and negative changes. Similarly, the positive effects of increased herbaceous vegetation could have offset and possibly overridden any negative effects of invasion on the microflora of *Quercus* sites.

The changes in soil properties and microbial biomass were also reflected in the abundance of microbial feeding nematodes, i.e., bacterivores and fungivores, but only in *Pinus* sites, where these trophic groups dominated. Microbial feeders accounted for 94% of the total nematode community at (Pc), while this percentage dropped to 71% at (Ppr) and further to 54% at Pinv. The reduced nematode abundance at Pinv could result from the allelopathic compounds of *S. elaeagnifolium*, which have exhibited nematicidal effects [51,52,69]. However, Karmezi et al. [54] did not report any suppression of microbivorous soil nematodes in the rhizospheres of *S. elaeagnifolium*. Besides, the herbivorous nematode groups that should be directly affected by the plant’s properties were not affected by disturbance and invasion at *Pinus* sites. Negative effects of plant invasion on microbivorous nematodes reported in other studies have been attributed either to the low quality of litter produced by the invasive plant [70] or to the plant’s allelochemicals [10], which both suppress and change primarily the community of decomposers and, consequently, the community of nematodes that feed on them. Thus, given the reduced values of C_org_, N_org_ and microbial biomass at Pinv, it seems more likely that in the case of pines, changes in the soil food web due to disturbance regimes and *S. eleagnifolium* invasion were bottom-up controlled.

Although, in pines, the changes in total nematode abundance reflected the changes in the microbial feeders, which were probably dictated by those of microbial biomass and soil properties, the pattern was not straightforward in the case of the Kermes oak shrublands. Previous studies [24] have suggested that although positive correlations between PLFAs and nematodes indicate bottom-up controlled food webs, this might be circumstantial and associated with certain soil factors, such as soil moisture, carbon, and nitrogen content. All the latter parameters exhibited lower values in *Quercus* sites than in *Pinus* sites. Indeed, in our *Quercus* sites, the relationships between soil biota and the changes due to the disturbance regime were more complicated. Unlike pines, the changes in both soil properties and microbial groups responded positively to invasion, but bacterivores and fungivores were not affected. However, there was a pronounced increase in plant parasites in the invaded site (Qinv), although this change was not statistically significant. Other studies [5] have also found different nematode responses related to *B. tectorum* invasion in two different grassland associations, but in both systems, plant feeders are reported to be almost absent, with the contrasting effects of invasion resulting from the different responses of bacterivores and fungivores to changes in their food resources. Regarding the responses of herbivores to invasions, both positive and negative responses have been reported in the literature [28,30], being attributed either to the well-developed root system of the focal invasive plant (*S. gigantea*) that could serve as a food source for herbivores or to the plant’s (*H. sosnowskyi*) allelochemicals making it less palatable. Previous research [54] demonstrated that *S. elaeagnifolium,* despite its allelochemical compounds, was more susceptible to root herbivory compared to an indigenous antagonistic plant during early invasion (10 years) and that this situation reversed only after the long naturalization of this alien plant (70 years). The *Quercus* sites studied herein supported more herbivores and fewer microbivores than the *Pinus* sites. The two herbivorous groups accounted for 35% at Qc, 39% at Qpr and 57% at Qinv. The increase in herbivores at Qinv might be the reason for the increase in microbial biomass at these sites since the infestation of plant roots by herbivorous and even more by plant parasitic nematodes increases leakage of root exudates, stimulating microbial growth [71]. In light of the above-mentioned, it seems that in Kermes oak shrublands, unlike pines, the soil food web is mostly regulated by the direct relationship of plant-feeders with the above-ground vegetation.

Apart from the Enrichment Index, nematode functional indices did not provide statistically significant results. Pines and shrublands could not be differentiated, because both the free-living and the plant-feeding dominant genera belonged to the cp-2 guild. Nevertheless, the indices offered an assessment of the prevailing conditions in both habitats, indicating stressed soils supporting degraded food webs, and having high fungal participation in the decomposition pathway [72,73,74,75]. Such conditions are not surprising, as urban and surrounding areas usually suffer from high and frequent anthropogenic pressures. Only EI, which is based on the abundance of the cp-1 enrichment opportunists, revealed the differences among the *Quercus* sites discussed above. In this study, only three genera of enrichment opportunists were found (Table 4). Among the *Quercus* sites, *Rhabditis* was found only at Qinv, where both *Mesorhabditis* and *Panagrolaimus* populations increased, thus raising the EI values. These nematodes responded to the increase in microbial biomass that was induced by vegetation via the activity of herbivores, indicating the cascading effects of below-ground herbivory on soil microbiota and microbial feeders, i.e., on the detritus food chain. On the other hand, these effects were probably not enough to induce analogous changes in the studied *Pinus* sites, where only the invasion of *S. elaeagnifolium* reduced the EI values.

Nematode communities with distinct genera, structure and diversity were formed as dictated by different habitat types and disturbance regimes (Table 3, Figure 3). Among the *Pinus* sites, the distinction between nematode communities derived mainly from the distinct assemblages of microbial feeders. The assemblages of the two microbial feeding groups in pines were shaped mainly by the responses of the bacterivore *Acrobeles* and the fungivore *Ditylenchus*, which both responded negatively to invasion. Their dominance at Pc was the main reason for the low diversity of that site. We should note that apart from the striking exception of *Chiloplacus*, the responses of the microbial feeding genera to the invasion of *S. elaeagnifolium* were more or less uniform in pines (Table 4), since the vast majority of their populations dropped at Pinv. Thus, bacterivorous and fungivorous populations changed in terms of magnitude rather than direction, resulting in distinct assemblages at the *Pinus* sites. As regards the *Quercus* sites, changes in communities due to the disturbance regime mainly refer to the varied responses of herbivorous genera. Changes in the herbivorous group were mainly shaped by the dominant *Paratylenchus*, an *r*-selected phytoparasite that has been reported to overdominate in ecosystems undergoing degradation, indicating alterations in vegetation cover [76]. Moreover, in exposed areas, where temperature and moisture fluctuations are less buffered by the canopy cover, *Paratylenchus* has a competitive advantage over other nematode genera [77]. Indeed, the population of *Paratylenchus* increased from the core to the peripheral and further to the invaded sites in both habitat types studied herein, while the overdominance of this genus at Qinv was responsible for the site’s low diversity. The responses of the other plant-feeding genera to disturbance and invasion were either positive, negative or neutral due to the variable food sources offered by the plants that colonized these sites. This resulted in distinct assemblages of plant feeders (Table 3 and Table 4). It is probably because of these mixed responses that the abundance of herbivores did not change statistically significantly among the *Quercus* sites (Figure 2). In both habitat types, the highest numbers of nematode genera were recorded in the peripheral sites that were not invaded by *S. elaeagnifolium* (Qpr and Ppr), probably because of the high number of native plant species that have colonized these areas (Table S4), offering a variety of resources to soil nematodes.

## 4. Materials and Methods

### 4.1. Study Area

Our sampling sites were maquis, more specifically kermes oak shrublands (*Quercus coccifera* L.) and pine forests (*Pinus brutia* Ten.) that were invaded by *S. elaeagnifolium*. They were located in the eastern part of the metropolitan area of the city of Thessaloniki in Northern Greece, where the presence of *S. elaeagnifolium* dates back to 1946 (or even earlier), although its invasion in the area was only reported after the 1970s [78] and in later times [79,80,81,82]. The climate is transient between Mediterranean and Continental, with a mean annual temperature 16.2 °C and a mean annual precipitation of 462 mm [83]. *S. elaeagnifolium* is considered to date to be one of the most abundant and widespread alien species in the urban and suburban areas of metropolitan Thessaloniki and one of the most noxious invasive plant species in Greece [38,39,40]. All sites were subject to anthropogenic disturbance, e.g., livestock grazing, proximity to residential areas and road networks.

### 4.2. Soil Sampling

The sampling scheme included sites that belonged to two different habitat types, namely *Q. coccifera* shrublands and *P. brutia* forests, but were also indicative of a different disturbance regime within each habitat type; the core of the shrublands or the forests were relatively undisturbed, dense formations, while the peripheral areas were subject to more intense anthropogenic pressure, resulting in open, bare soil spaces sparsely covered by several weeds (Table S4). This degradation may have facilitated the invasion of *S. elaeagnifolium*, an additional pressure leading to even greater habitat degradation [54,84,85]. In the invaded peripheral areas, the coverage of *S. elaeagnifolium* reached 30–50%. Four *Quercus* shrublands (Q) and four *Pinus* forested sites (P) were selected for soil sampling (2 habitat types × 4 replicate sites). In each one of the eight sites, one composite soil sample was taken from the relatively non-disturbed core of the formation (Qc and Pc), one from its degraded peripheral areas that were invaded yet by *S. elaeagnifolium* (Qpr and Ppr), and one from the degraded peripheral areas that have been invaded by *S. elaeagnifolium* (Qinv and Pinv). Each composite soil sample consisted of five soil cores (7.5 cm diameter, 20 cm depth), taken from the open spaces close to the Kermes oak shrubs or the pine trees. A total of 24 samples (2 habitat types × 3 disturbance regimes × 4 replicate sites) were collected, transferred and stored at 4 °C in the laboratory, followed by soil analyses and nematode extraction.

### 4.3. Soil Physicochemical Properties

Promptly after sampling, a part of the collected soil samples was sieved (mesh size 2 mm) and air-dried and used for the determination of soil texture, water content (%), pH and soil organic C and N. Soil texture was estimated by the Bouyoucos hydrometer method [86], and pH was measured using an electrode pH-meter in a 1:2 *w*/*v* soil:water suspension [87]. For the estimation of soil organic C (C_org_), initially soil organic matter (SOM_loi_) was determined by the Loss on Ignition (LOI) method at 375 °C for 16 h, after soil samples were initially dried at 105 °C for 24 h [88], and soil organic C (SOC_loi_) was estimated according to Jensen et al. [89]. Soil organic N (N_org_) was measured by the Kjeldahl method [90].

### 4.4. Phospholipid Fatty Acid Analysis (PLFA) Extraction and Classification

For the extraction of phospholipid fatty acids (PLFAs), we took a subsample of 5 g dry weight from each composite soil sample. The extraction was performed as described in brief in Monokrousos et al. [91], whereas a more detailed account of the extraction is presented in Spyrou et al. [92]. The procedure is described in brief as follows: (i) extraction of lipids; (ii) separation of phospholipids by column chromatography; (iii) methylation of esterified fatty acids in the phospholipid fraction; and (iv) chromatographic separation and identification of the main components on a Trace GC Ultra gas chromatograph (GC) (Thermo-Finnigan, San Jose, CA, USA) coupled with a Trace ISQ mass spectrometry detector, a split−splitless injector and an Xcalibur MS platform [93]. Fatty acids were quantified (nmol g^−1^) by calibration against standard solutions of the internal standard 19:0 ME. For this, a six-point calibration curve was constructed in the range of 25–200 g mL^−1^ 19:0 ME. Under the above-described conditions, the GC response to 19:0 ME was linear in the range of 25–200 g mL^−1^, with acceptable recoveries. For the classification of phospholipid fatty acids, the retention times of the individual peaks were compared with those obtained from the commercial standard mixtures FAME and BAME (47885-U and 47080-U, respectively; Supelco, Nottingham, UK) and was performed with the Thermo Xcalibur 2.2 software [93].

The recovered PLFA signature biomarkers were then assigned into the following functional groups [94,95,96,97,98,99,100,101,102]: a15:0, i15:0, i16:0 and i17:0 for Gram-positive bacteria (G+); 2-OH10:0, 2-OH 12:0, 3-OH 12:0, 2-OH 14:0, 3-OH 14:0, 2-OH16:0, 16:1ω7c, 17:1ω7c, cy17:0 and cy19:0 for Gram-negative bacteria (G−); 10Me16:0, 10Me17:0 and 10Me18:0 for actinobacteria. In addition, 18:2ω6c and 18:3ω6c biomarkers were considered to be of fungal origin; 20:2ω6c, 20:3ω6c and 20:4ω6c were assigned to protozoa; 20:5ω3c 22:6ω3c, 22:0, 23:0 and 24:0 were characterized as general microeukaryotic biomarkers; 15:0 and 17:0 signature biomarkers were considered to be of bacterial origin, while 14:0, 18:0 and 20:0 were of microbial origin; 16:0 biomarker was assigned to both bacteria and fungi, while 18:1ω9c and 18:1ω9t biomarkers were assigned to both Gram-negative bacteria and fungi.

### 4.5. Nematode Extraction and Identification

From each composite soil sample, we took a subsample of 200 mL soil to extract nematodes. Prior to extraction, each soil sample was carefully mixed by hand, and soil clusters were gently broken up. Cobb’s modified sieving and decanting method was used for the extraction [103]. After extraction, living nematodes were counted under a stereomicroscope, and then they were heat-killed and fixed with a 4% formaldehyde solution. From each soil sample, 100 randomly selected nematodes were identified to genus level [104]. Each genus was further assigned to trophic groups [105] and classified across the colonizer-persister scale (cp values) [106,107].

### 4.6. Nematode Functional Indices

The Maturity Index (MI) for free-living nematodes and the Plant Parasitic Index (PPI) for plant-feeding nematodes, which both indicate the successional status of the community, were calculated according to Bongers [72]. The Enrichment index (EI), the Channel Index (CI) and the Structure Index (SI), which indicate the functional structure of the food web, were calculated according to the weighted faunal analysis proposed by Ferris et al. [74]. EI indicates soil enrichment with organic material, mirroring the increases in enrichment opportunistic nematodes, mainly bacterial feeders, which respond rapidly to increases in food. SI is an indicator of long and complex soil food webs with high connectance and numerous trophic links, weighting the prevalence of omnivores and predatory nematodes. Finally, CI indicates the degree of fungal participation in the decomposition channel of the soil food web [74].

### 4.7. Data Analysis

To evaluate the effects of habitat type (Q: *Quercus*, P: *Pine*) and disturbance regime (c: core, pr: periphery non-invaded, inv: periphery invaded) on soil variables, nematodes and microbial groups, we used Permutational Multivariate Analysis of Variance (PERMANOVA; [108]). All PERMANOVA analyses were performed with “habitat” (Q, P) as a fixed factor and “disturbance” (c, pr, inv) nested within the factor “habitat”. Pair-wise *a posteriori* tests were performed among levels of the factor “disturbance” within the factor “habitat”. The analysis performed 4999 permutations. PERMANOVA was performed on (i) single variables, i.e., soil properties, PLFA functional groups, total nematode abundance, abundance of individual nematode trophic groups and nematode indices using the Euclidean distance measure, as well as on (ii) multivariate datasets, that is, the entire ensemble of nematode genera and the genera ensemble of each nematode trophic group using the Bray–Curtis measure. For these analyses, we used the Fortran software PERMANOVA [108].

For assessing the diversity of nematode communities, we used the method of diversity ordering described by Patil and Taillie [109], which is based on Renyi’s index [110]. Renyi’s parametric index of order α shows varying sensitivity to the rare and abundant species of a community as the scale parameter α changes [111]. For each community, it provides a profile of the most widely used diversity indices. For α = 0, the index equals the total number of species; for α = 1, it equals Shannon’s index; for α = 2, it equals Simpson’s index. For α tending to infinite, the index is most sensitive to the abundant species in a community. Thus, when two diversity profiles differ in the range of low α values, this is due to the number of species. In the range of high α values, differences between communities are due to the presence of abundant species. When two diversity profiles intersect, the two communities may be ordered differently by different diversity indices [112]. In our study, nematode genera instead of species were used, and calculations were performed with Past 3.17 [113].

## 5. Conclusions

Most studied variables were affected by habitat type, while the effect of invasion by *S. elaeagnifolium* was different in each habitat. Compared to the *Quercus coccifera* shrublands, the soil in *Pinus brutia* forests was “richer”, with a higher silt content and a lower sand content, higher water and organic content and a much larger microbial biomass and abundance of microbial feeding nematodes, indicating a bottom-up control of the detritus food web. The invasion of *S. elaeagnifolium* in pines had a negative effect on soil properties and microbial biomass, which was reflected in most bacterivorous and fungivorous genera. No direct effect of the invasion on nematodes was revealed. On the contrary, in the maquis shrublands, both soil properties and microbial biomass responded positively to invasion, but this was probably caused by the plants that colonized the disturbed peripheral areas of the shrublands and offered a qualitative food source to microbes and root herbivores, mostly *Paratylenchus*. Root herbivory probably increased root leakage, further enhancing microbial growth. Thus, in the “poor” maquis shrublands, soil enrichment and microbial enhancement due to colonizing plants and root herbivory were so important that they could have overridden any negative effects of invasion but were not enough to impose any significant change in the “rich” soil of pines. We conclude that in pines, the main driver of changes in the soil food web of disturbed areas was the negative effect of *S. elaeagnifolium* on microbial biomass, while in maquis, changes in the soil food web were mostly driven by plants and root herbivores.

Undoubtedly, more investigations and experimental data are needed to unveil the complex phenomenon of plant invasions globally, involving more invasive plants and more ecosystems. However, our results on the potential underlying mechanisms of *S. elaeagnigolium* invasiveness will contribute to future targeted management strategies against its ongoing invasion across the world.

## Figures and Tables

**Table 1:** Soil physicochemical properties (mean ± SE) in two different habitat types (*Quercus coccifera* shrublands, *Pinus brutia* forests) and three different disturbance regimes (c: core, pr: periphery not invaded, inv: periphery invaded by *Solanum elaeagnifolium*). PERMANOVA results for the effects of “habitat” (Hab) and “disturbance” (Dist) are shown (“Dist” nested within “Hab”) (ns: non-significant, * *p* < 0.05, ** *p* < 0.01, *** *p* < 0.001). Different letters a, b indicate significant differences revealed by pair-wise comparisons between disturbance regimes within each habitat type. For all cases *n* = 4.

Soil Properties	*Quercus coccifera*			*Pinus brutia*			Hab	Dist
	Qc	Qpr	Qinv	Pc	Ppr	Pinv		
Clay (%)	5.75 (1.14) ^a^	9.11 (0.59) ^b^	9.93 (1.45) ^ab^	8.52 (1.27) ^ab^	7.11 (0.45) ^a^	11.61 (1.00) ^b^	ns	*
Silt (%)	33.37 (1.35) ^a^	22.36 (0.90) ^b^	29.68 (2.69) ^ab^	30.27 (2.46)	35.18 (2.14)	34.75 (0.77)	*	**
Sand (%)	60.89 (1.88) ^a^	68.53 (1.48) ^b^	60.39 (2.48) ^a^	61.21 (2.59) ^a^	57.71 (2.14) ^ab^	53.64 (0.91) ^b^	**	*
Water content (%)	2.91 (0.82)	2.22 (0.65)	2.22 (0.20)	2.99 (0.44)	3.81 (0.51)	3.38 (0.28)	*	ns
pH	6.39 (0.05)	6.44 (0.06)	6.25 (0.12)	6.28 (0.10)	6.33 (0.13)	6.49 (0.09)	ns	ns
Organic C (%)	1.65 (0.29)	1.51 (0.27)	1.79 (0.27)	4.92 (0.98) ^ab^	4.68 (0.69) ^a^	2.64 (0.17) ^b^	***	ns
Organic N (%)	0.15 (0.02) ^ab^	0.11 (0.02) ^a^	0.24 (0.04) ^b^	0.37 (0.07)	0.41 (0.08)	0.26 (0.03)	***	ns
C_org_/N_org_	11.55 (1.34) ^ab^	14.21 (1.44) ^a^	7.68 (1.05) ^b^	13.44 (1.03)	11.84 (0.89)	10.62 (1.09)	ns	**

**Table 2:** Mean values (±SE) of total nematode abundance (individuals per 100 g dry soil) and nematode functional indices in two different habitat types (*Quercus coccifera* shrublands, *Pinus brutia* forests) and three different disturbance regimes (c: core, pr: periphery not invaded, inv: periphery invaded by *Solanum elaeagnifolium*). PERMANOVA results for the effects of “habitat” (Hab) and “disturbance” (Dist) are shown (“Dist” nested within “Hab”) (ns: non-significant, * *p* < 0.05). Different letters a, b indicate significant differences revealed by pair-wise comparisons between disturbance regimes within each habitat type. For all cases *n* = 4.

	*Quercus coccifera*			*Pinus brutia*			Hab	Dist
	Qc	Qpr	Qinv	Pc	Ppr	Pinv		
Maturity Index	2.07 (0.06)	2.15 (0.08)	1.97 (0.05)	2.05 (0.02)	2.08 (0.06)	2.02 (0.01)	ns	ns
Plant Parasitic Index	2.19 (0.06)	2.15 (0.03)	2.11 (0.08)	2.43 (0.20)	2.04 (0.04)	2.14 (0.09)	ns	ns
Structure Index	14.81 (8.72)	30.90 (8.38)	16.11 (6.65)	15.47 (2.96)	19.44 (8.37)	9.86 (1.50)	ns	ns
Enrichment Index	34.10 (2.81) ^a^	38.60 (5.30) ^ab^	47.50 (4.0) ^b^	38.05 (1.53) ^a^	40.36 (1.58) ^a^	31.27 (1.6) ^b^	ns	*
Channel Index	84.00 (9.24)	74.22 (9.25)	46.42 (15.77)	74.84 (9.35)	76.23 (8.67)	75.34 (6.21)	ns	ns

**Table 3:** PERMANOVA results for the effects of factors “habitat” and “disturbance” (within factor “habitat”) on multiple variable data sets, i.e., abundances of nematode genera (ns: non-significant, * *p* < 0.05, ** *p* < 0.01, *** *p* < 0.001). Significant differences revealed by pair-wise comparisons between disturbance regimes within each habitat type are indicated.

	Habitat	Disturbance	Pair–Wise Tests
All genera	***	***	Qpr ≠ Qinv * Pc ≠ Pinv * Ppr ≠ Pinv **
Root/fungal feeding genera	***	**	Qc ≠ Qinv * Qpr ≠ Qinv *
Plant parasitic genera	**	*	
Bacterivorous genera	**	**	Ppr ≠ Pinv *
Fungivorous genera	**	**	Pc ≠ Pinv * Ppr ≠ Pinv **
Predatory genera	ns	ns	
Omnivorous genera	ns	ns	

**Table 4:** Mean abundance (Ab: individuals per 100 g dry soil) of nematode genera and percentage participation to the total community (Part) in two different habitat types (Q: *Quercus coccifera* shrublands, P: *Pinus brutia* forests) and three different disturbance regimes (c: core, pr: periphery not invaded, inv: periphery invaded by *Solanum elaeagnifolium*). The colonizer-persister value (c-p) of each genus is also indicated.

Genus	c-p	Qc		Qpr		Qinv		Pc		Ppr		Pinv	
		Ab.	Part.	Ab.	Part.	Ab.	Part.	Ab.	Part.	Ab.	Part.	Ab.	Part.
Root/fungal feeders													
*Boleodorus*	2	74.58	5.03	1.82	0.19	27.20	1.86			44.17	2.41	20.12	2.63
*Filenchus*	2	232.42	15.68	197.45	20.40	36.40	2.49	76.75	1.85	141.62	7.73	17.03	2.22
*Malenchus*	2	31.05	2.10	37.74	3.90	147.17	10.06			11.34	0.62	16.76	2.19
*Tylenchus*	2	14.06	0.95	9.75	1.01	18.62	1.27			13.11	0.72	3.28	0.43
Parasitic plant feeders													
*Bitylenchus*	3	8.68	0.59	1.82	0.19	8.33	0.57						
*Helicotylenchus*	3	10.84	0.73	5.08	0.53	6.13	0.42	3.37	0.08				
*Hemicycliophora*	3			27.90	2.88			117.57	2.83				
*Heterodera*	3					4.15	0.28						
*Meloidogyne*	3											4.69	0.61
*Merlinius*	3	25.72	1.74			19.61	1.34			5.52	0.30		
*Paratylenchus*	2	85.59	5.78	86.93	8.98	543.55	37.15	14.38	0.35	47.39	2.59	87.12	11.37
*Pratylenchus*	3					7.28	0.50			11.04	0.60	1.27	0.17
*Pungentus*	4	8.57	0.58	1.82	0.19			17.27	0.42				
*Scutylenchus*	3	44.97	3.04	11.57	1.20	7.60	0.52					9.11	1.19
*Trichodorus*	4	4.34	0.29	2.90	0.30								
Bacterivores													
*Acrobeles*	2	168.61	11.38	41.94	4.33	45.32	3.10	954.61	22.96	101.53	5.54	60.61	7.91
*Acrobeloides*	2	120.06	8.10	32.57	3.37	119.78	8.19	300.32	7.22	162.02	8.84	71.87	9.38
*Cervidellus*	2	57.63	3.89	38.81	4.01	68.37	4.67	289.24	6.96	82.82	4.52	31.24	4.08
*Chiloplacus*	2	3.42	0.23	21.68	2.24	18.87	1.29	53.93	1.30	43.41	2.37	133.64	17.44
*Chronogaster*	3			2.90	0.30							1.27	0.17
*Drilocephalobus*	2					12.46	0.85						
*Eucephalobus*	2	84.43	5.70	65.44	6.76	37.93	2.59	165.90	3.99	44.09	2.41	35.15	4.59
*Eumonhystera*	2	3.42	0.23	3.27	0.34			63.09	1.52	144.24	7.87	2.54	0.33
*Geomonhystera*	2									24.16	1.32		
*Mesorhabditis*	1	2.17	0.15	1.82	0.19	18.40	1.26	235.13	5.65	14.20	0.78	1.27	0.17
*Monhystera*	2			2.44	0.25					5.10	0.28		
*Panagrolaimus*	1	9.01	0.61	22.09	2.28	56.43	3.86	17.10	0.41	39.75	2.17	16.92	2.21
*Plectus*	2	10.77	0.73	26.29	2.72	20.77	1.42	218.41	5.25	49.11	2.68	7.13	0.93
*Prismatolaimus*	3	20.51	1.38	10.58	1.09			8.64	0.21				
*Rhabditis*	1					14.44	0.99	3.37	0.08	8.43	0.46		
*Rhabdolaimus*	3	3.42	0.23	7.27	0.75					26.04	1.42		
*Teratocephalus*	3			19.06	1.97			73.88	1.78	39.93	2.18		
*Wilsonema*	2	49.83	3.36	28.40	2.93	24.73	1.69	113.39	2.73	50.19	2.74	42.41	5.53
Fungivores													
*Aphelenchoides*	2	181.94	12.28	69.25	7.16	36.39	2.49	367.46	8.84	184.41	10.07	67.11	8.76
*Aphelenchus*	2	26.16	1.77	15.96	1.65	47.51	3.25	159.79	3.84	72.51	3.96	62.92	8.21
*Diphtherophora*	3			5.45	0.56								
*Ditylenchus*	2	240.94	16.26	141.23	14.59	84.80	5.80	765.71	18.41	424.24	23.16	57.19	7.46
*Funaria*	4					2.08	0.14						
*Tylencholaimellus*	4					12.46	0.85	131.58	3.16	23.29	1.27		
*Tylencholaimus*	4	10.26	0.69	6.16	0.64	2.08	0.14						
Predators													
*Aporcelaimellus*	5	16.15	1.09	9.43	0.97	4.15	0.28						
*Aporcelaimus*	5									2.91	0.16		
*Discolaimus*	5							3.37	0.08				
*Eudorylaimus*	4	3.42	0.23					4.32	0.10	15.29	0.83	13.22	1.72
*Prionchulus*	4			9.09	0.94								
*Thonus*	4	3.42	0.23	1.82	0.19								
Omnivores													
*Microdorylaimus*	4					10.29	0.70					2.54	0.33

## Data Availability

Data available on request.

## Supplementary Materials

The following supporting information can be downloaded at: https://www.mdpi.com/article/10.3390/plants12112193/s1, Table S1: Mean concentration (±SE) of PLFA biomarkers (nmoles/g) recovered in two different habitat types (Q: *Quercus coccifera* shrublands, P: *Pinus brutia* forests) and three different disturbance regimes (c: core, pr: periphery not invaded, inv: periphery invaded by *Solanum elaeagnifolium*); Table S2. Biomass of microbial groups (nmol/g) in each composite sample taken from the four replicate sites of two different habitat types (Q: *Quercus coccirefa* shrublands, P: *Pinus brutia* forests) and three different disturbance regimes (c: core, pr: periphery not invaded, inv: periphery invaded by *Solanum elaeagnifolium*). Table S3. Abundance of nematode trophic groups in each composite sample taken from the four replicate sites of two different habitat types (Q: *Quercus coccirefa* shrublands, P: *Pinus brutia* forests) and three different disturbance regimes (c: core, pr: periphery not invaded, inv: periphery invaded by *Solanum elaeagnifolium*).Table S4: List of Greek native plant species and subspecies found in *Quercus coccifera* shrublands (Q) and *Pinus brutia* forests (P) at three different disturbance regimes (c: core, pr: periphery not invaded, inv: periphery invaded by *Solanum elaeagnifolium*). The presence of plants is indicated by colored cells.
