# Supplementary material for: The Invasion of Alien Populations of Solanum elaeagnifolium in Two Mediterranean Habitats Modifies the Soil Communities in Different Ways
Source: Plants (Basel). 2023 May 31;12(11):2193. doi: 10.3390/plants12112193 (PMC10255462; doi:10.3390/plants12112193)
Supplement: Supplementary file 1 [file plants-12-02193-s001.zip › plants-2409719-supplementary.pdf]

Supplementary material

The Invasion of Alien Populations of *Solanum elaeagnifolium* in Two Mediterranean Habitats Modifies the Soil Communities in Different Ways

Maria Karmezi [1,*], Nikos Krigas [2], Efimia M. Papatheodorou [3] and Maria D. Argyropoulou [1,*]

[1] Department of Zoology, School of Biology, Faculty of Sciences, Aristotle University, 54124 Thessaloniki, Greece
[2] Institute of Plant Breeding and Genetic Resources, Hellenic Agricultural Organization Demeter, 57001 Thessaloniki, Greece; nikoskrigas@gmail.com
[3] Department of Ecology, School of Biology, Faculty of Sciences, Aristotle University, 54124 Thessaloniki, Greece; papatheo@bio.auth.gr
* Correspondence: mkarmezi@bio.auth.gr (M.K.); margyrop@bio.auth.gr (M.D.A.)

s

Table S1. Mean concentration (± SE) of PLFA biomarkers (nmol/g) recovered in two different habitat types (Q: *Quercus coccirefa* shrublands, P: *Pinus brutia* forests) and three different disturbance regimes (c: core, pr: periphery not invaded, inv: periphery invaded by *Solanum elaeagnifolium*).

PLFA biomarkes	Functional groups	Qc	Qpr	Qinv	Pc	Ppr	Pinv
2-OH10:0	G-				0.16 (0.10)	0.18 (0.11)	
2-OH12:0	G-		0.44 (0.25)	0.29 (0.18)	0.41 (0.05)	0.55 (0.08)	0.49 (0.06)
3-OH12:0	G-				0.71 (0.35)	0.28 (0.19)	0.16 (0.16)
14:0	Microbia	3.93 (0.73)	4.95 (0.37)	5.40 (1.46)	22.66 (1.54)	23.78 (0.90)	14.88 (1.51)
i15:0	G+	18.35 (2.82)	20.36 (1.70)	24.75 (4.93)	32.19 (1.42)	32.86 (0.85)	29.25 (1.79)
a15:0	G+	11.09 (1.82)	14.05 (1.44)	16.20 (3.18)	24.39 (2.21)	25.68 (0.30)	20.48 (2.02)
15:0	Bacteria	2.35 (0.56)	2.34 (0.29)	2.48 (0.98)	7.54 (0.94)	7.71 (0.61)	4.81 (0.49
2-OH14:0	G-			0.03 (0.03)		0.80 (0.80)	
3-OH14:0	G-		0.32 (0.19)	0.60 (0.36)	0.97 (0.21)	2.30 (1.58)	1.12 (0.60)
i16:0	G+	10.65 (1.27)	12.17 (1.14)	13.39 (2.79)	22.43 (1.43)	17.20 (5.75)	18.92 (1.89)
16:1ω7c	G-	11.45 (1.76)	12.43 (1.51)	14.71 (3.01)	22.02 (1.07)	21.75 (0.20)	15.80 (5.37)
16:0	Bacteria/fungi	28.55 (1.82)	28.97 (3.33)	33.17 (4.65)	38.77 (4.04)	40.01 (0.36)	35.43 (1.38)
10Me16:0	Actinobacteria	7.29 (1.10)	7.72 (0.97)	9.90 (2.17)	13.30 (0.66)	12.30 (0.82)	11.20 (1.01)
i17:0	G+	5.42 (0.54)	6.09 (0.66)	7.09 (1.57)	11.95 (1.09)	12.18 (0.61)	8.82 (0.75)
17:1ω7c	G-	2.52 (0.34)	2.85 (0.21)	3.95 (1.28)	7.81 (1.17)	8.84 (0.67)	5.49 (0.55)
cy17:0	G-	6.99 (0.78)	7.74 (0.87)	4.93 (1.82)	16.82 (1.22)	15.28 (0.91)	12.68 (1.20)
17:0	Bacteria	4.62 (0.48)	5.41 (0.44)	6.01 (1.22)	8.52 (0.91)	8.68 (0.47)	8.21 (1.09)
2-OH16:0	G-				0.05 (0.05)		0.47 (0.47)
10Me17:0	Actinobacteria	1.48 (0.21)	1.88 (0.23)	1.92 (0.39)	4.25 (0.48)	4.66 (0.50)	2.76 (1.02)
18:3ω6c	Fungi	0.40 (0.16)	0.70 (0.11)	1.03 0.42)	1.96 (0.24)	2.25 (0.13)	1.70 (0.47)
18:2ω6c	Fungi	6.96 (0.55)	7.35 (0.97)	7.52 (1.46)	18.33 (1.08)	15.64 (0.72)	12.52 (0.59)
18:1ω9c	G-/fungi	16.86 (1.36)	17.91 (1.52)	20.07 (3.41)	24.60 (1.41)	25.54 (0.49)	22.64 (1.25)
18:1ω9t	G-/fungi	14.80 (1.82)	15.07 (1.76)	17.61 (3.32)	25.87 (2.48)	24.03 (2.15)	20.96 (1.67)
18:0	Microbia	8.76 (0.95)	9.97 (1.19)	11.68 (2.45)	19.62 (2.71)	20.66 (0.34)	18.05 (1.98)
10Me18:0	Actinobacteria	5.17 (0.80)	6.29 (0.94)	6.79 (1.33)	10.93 (0.88)	12.01 (1.08)	10.08 (1.39)
cy19:0	G-	0.87 (0.17)	1.22 (0.31)	1.46 (0.52)	2.51 (0.28)	3.12 (0.22)	1.58 (0.15)
20:4ω6c	Protozoa	0.19 (0.05)	0.30 (0.04)	0.40 (0.12)	0.06 (0.06)		0.66 (0.41)
20:5ω3c	Micro-eukaryotes	0.23 (0.10)	0.25 (0.07)	0.34 (0.10)	0.46 (0.09)	0.57 (0.09)	0.53 (0.09)
20:3ω6c	Protozoa	0.79 (0.22)	0.93 (0.19)	1.30 (0.56)	1.92 (0.32)	2.66 (0.35)	2.16 (0.56)
20:2ω6c	Protozoa	0.69 (0.07)	0.64 (0.04)	0.69 (0.09)	0.62 (0.06)	0.67 (0.06)	0.73 (0.02)
20:0	Microbia	1.91 (0.38)	1.93 (0.21)	2.40 (0.78)	7.88 (0.64)	10.43 (0.55)	5.68 (0.57)
22:6ω3c	Micro-eukaryotes	2.17 (0.64)	1.87 (0.43)	2.10 (0.53)	3.32 (0.43)	2.84 (0.40)	4.29 (0.45)

PLFA biomarkes	Functional groups	Qc	Qpr	Qinv	Pc	Ppr	Pinv
22:0	Micro-eukaryotes	1.45 (0.19)	1.70 (0.07)	2.14 (0.71)	8.78 (0.84)	12.80 (0.74)	5.32 (0.68)
23:0	Micro-eukaryotes	0.17 (0.03)	0.15 (0.01)	0.25 (0.08)	0.53 (0.20)	1.07 (0.08)	0.45 (0.04)
24:0	Micro-eukaryotes	0.55 (0.12)	0.58 (0.03)	0.71 (0.22)	5.00 (0.62)	6.82 (0.56)	2.02 (0.18)

Table S2. Biomass of microbial groups (nmol/g) in each composite sample taken from the four replicate sites of two different habitat types (Q: *Quercus coccirefa* shrublands, P: *Pinus brutia* forests) and three different disturbance regimes (c: core, pr: periphery not invaded, inv: periphery invaded by *Solanum elaeagnifolium*).

Samples	Microbia	Bacteria	G+	G-	Actinobacteria	Fungi	Protozoa	Microeukaryotes
**Qc1**	153.57	77.92	40.12	18.12	14.85	5.66	1.30	7.21
**Qc2**	232.63	121.53	63.23	30.70	19.13	9.12	2.32	6.88
**Qc3**	165.34	84.03	45.52	20.22	11.82	7.25	1.59	5.39
**Qc4**	152.37	66.10	33.19	18.28	9.96	7.40	1.49	5.46
**Qpr1**	182.17	97.93	49.85	24.02	18.43	7.18	1.50	6.92
**Qpr2**	218.83	114.44	61.37	28.97	17.38	7.90	1.85	5.74
**Qpr3**	226.73	114.82	59.33	29.72	17.72	11.01	2.00	5.67
**Qpr4**	148.10	73.17	40.12	17.28	10.06	6.13	2.13	7.37
**Qinv1**	135.31	65.81	35.32	16.25	11.52	5.49	1.28	6.61
**Qinv2**	162.00	83.43	45.62	19.14	12.64	5.25	1.44	5.36
**Qinv3**	302.25	157.67	84.96	38.67	23.99	12.31	3.33	10.26
**Qinv4**	282.84	145.19	79.80	29.76	26.29	11.15	3.50	9.47
**Pc1**	400.60	204.00	100.47	59.33	28.33	21.91	2.40	19.57
**Pc2**	361.39	174.01	85.00	48.43	29.70	22.08	2.00	22.36
**Pc3**	295.08	154.03	77.10	42.58	23.56	17.06	2.60	15.98
**Pc4**	409.77	205.75	101.25	55.52	32.33	20.12	3.40	24.83
**Ppr1**	379.83	188.60	92.00	52.71	30.30	18.14	3.00	25.34
**Ppr2**	360.52	177.84	94.14	47.62	24.18	16.05	3.73	26.74
**Ppr3**	396.54	196.59	92.37	53.56	34.82	19.37	2.69	29.53
**Ppr4**	365.03	170.00	73.18	58.50	26.56	18.01	3.89	28.13
**Pinv1**	300.78	150.86	73.74	43.49	21.91	14.41	4.26	15.83
**Pinv2**	343.48	189.04	95.09	48.83	30.24	15.18	2.87	17.05
**Pinv3**	258.60	132.52	65.16	38.02	19.98	12.42	3.61	14.41
**Pinv4**	295.48	130.54	75.86	20.83	24.02	14.86	3.43	17.28

Table S3. Abundance of nematode trophic groups in each composite sample taken from the four replicate sites of two different habitat types (Q: *Quercus coccirefa* shrublands, P: *Pinus brutia* forests) and three different disturbance regimes (c: core, pr: periphery not invaded, inv: periphery invaded by *Solanum elaeagnifolium*).

Samples	Total abundance	Root/ fungal feeders	Plant parasitic	Bacterivores	Fungivores	Predators	Omnivores
**Qc1**	871.17	260.26	234.24	229.19	147.48	0.00	0.00
**Qc2**	1367.63	205.14	13.68	847.93	273.53	27.35	0.00
**Qc3**	2243.66	571.92	384.02	474.53	813.19	0.00	0.00
**Qc4**	1743.08	371.13	122.93	581.44	602.97	64.60	0.00
**Qpr1**	1050.43	312.85	208.57	274.11	243.33	11.59	0.00
**Qpr2**	727.19	189.07	138.17	210.89	145.44	43.63	0.00
**Qpr3**	1158.67	182.93	117.60	434.23	397.79	26.13	0.00
**Qpr4**	934.55	302.17	87.73	378.95	165.71	0.00	0.00
**Qinv1**	2203.49	318.88	1099.27	564.18	196.63	0.00	24.53
**Qinv2**	1606.81	265.86	336.48	788.47	182.78	16.62	16.62
**Qinv3**	668.03	222.85	112.17	149.85	183.16	0.00	0.00
**Qinv4**	1374.81	109.99	838.64	247.47	178.73	0.00	0.00
**Pc1**	12913.80	155.46	470.27	8138.73	4149.35	0.00	0.00
**Pc2**	688.85	34.44	6.89	247.99	399.53	0.00	0.00
**Pc3**	1304.42	13.48	64.11	754.97	458.38	13.48	0.00
**Pc4**	1727.17	103.63	69.09	846.31	690.87	17.27	0.00
**Ppr1**	1214.17	163.05	69.88	398.92	570.67	11.65	0.00
**Ppr2**	2058.64	331.24	110.41	888.26	728.73	0.00	0.00
**Ppr3**	1798.55	346.65	40.78	595.48	754.47	61.17	0.00
**Ppr4**	2256.07	0.00	34.72	1457.41	763.93	0.00	0.00
**Pinv1**	554.12	20.34	40.68	340.54	142.39	0.00	10.17
**Pinv2**	804.90	112.69	201.23	321.96	160.98	8.05	0.00
**Pinv3**	636.87	63.69	6.37	343.91	210.17	12.74	0.00
**Pinv4**	1069.84	32.10	160.48	609.81	235.37	32.10	0.00

Table S4. List of Greek native plant species and subspecies found in *Quercus coccifera* shrublands (Q) and *Pinus brutia* forests (P) at three different disturbance regimes (c: core, pr: periphery not invaded, inv: periphery invaded by *Solanum elaeagnifolium*). The presence of plants is indicated by colored cells.

	Qc	Qpr	Qinv	Pc	Ppr	Pinv
Acinos alpinus (L.) Moench subsp. *nomismophyllus* (Rech. fil.) Leblebici	x	x	x			
Acinos suaveolens (Sm.) Loudon					x	
Alkanna tinctoria Tausch subsp. *tinctoria*				x	x	
Allium rhodopeum Velen.	x	x				
Allium sphaerocephalon L. subsp. *sphaerocephalon*	x	x	x			
Alyssum simplex Rudolphi		x	x			
Anacamptis pyramidalis (L.) Rich.	x	x	x			
Anthyllis hermanniae L. subsp. *hermanniae*		x				
Asparagus aphyllus L. subsp. *aphyllus*					x	
Avena barbata Link				x	x	
Ballota nigra L.						x
Brachypodium sylvaticum (Huds.) P. Beauv. subsp. *sylvaticum*		x	x			x
Bromus madritensis L.				x	x	
Celtis australis L.					x	
Centaurea grisebachii (Nyman) Heldr. subsp. *grisebachii*	x	x	x		x	
Chrysopogon gryllus (L.) Trin.	x	x	x			
Cistus creticus L. subsp. *creticus*		x	x	x	x	
Convolvulus althaeoides L.					x	x
Crupina crupinastrum (Moris) Vis.				x	x	
Cupressus sempervirens L.					x	
Cynosurus echinatus L.		x	x			
Dactylis glomerata subsp. *hispanica* (Roth) Nyman		x	x			
Dasypyrum villosum (L.) P. Candargy					x	
Dianthus corymbosus Sm.	x	x	x			
Dianthus illyricus (Ard.) Fassou, N.Korotkova, Dimop. & Borsch						x
Dianthus illyricus (Ard.) Fassou, N.Korotkova, Dimop. & Borsch subsp. *illyricus*		x			x	
Dianthus illyricus subsp. *haynaldianus* (Nyman) Fassou, N.Korotkova, Dimop. & Borsch	x	x				x
Dianthus pinifolius Sm. subsp. *pinifolius*					x	
Echium plantagineum L.				x	x	
Eryngium campestre L.	x	x	x			
Erysimum crassistylum C. Presl	x	x	x			
Festuca valesiaca Gaudin	x	x	x			x
Fumana thymifolia (L.) Webb						x
Helianthemum salicifolium (L.) Mill.	x	x				
Hirschfeldia incana (L.) Lagr.-Foss.		x	x		x	
Hypericum olympicum L.	x	x	x			
Hypericum perforatum L.					x	
Juniperus oxycedrus subsp. *deltoides* (R.P. Adams) N.G. Passal.	x	x	x	x	x	
Malabaila aurea (Sm.) Boiss.	x	x	x			
Melica ciliata L. subsp. *ciliata*		x	x			
Melilotus albus Medik.	x	x	x			
Micromeria juliana (L.) Rchb.					x	
Minuartia attica (Boiss. & Spruner) Vierh. subsp. *attica*	x	x	x			
Nigella arvensis L.	x					
Onopordum illyricum subsp. *cardunculus* (Boiss.) Arènes				x		

	**Qc**	**Qpr**	**Qinv**	**Pc**	**Ppr**	**Pinv**
Phillyrea latifolia L.		■				
Piptatherum miliaceum (L.) Coss.						■
Piptatherum miliaceum (L.) Coss. subsp. *miliaceum*	■	■	■			
Prunus spinosa subsp. *dasyphylla* (Schur) Domin	■	■	■			
Rostraria cristata (L.) Tzvelev						■
Salvia verbenaca L.					■	
Sarcopoterium spinosum (L.) Spach						■
Scabiosa triniifolia Friv.				■	■	■
Silene gigantea subsp. *rhodopea* (Janka) Greuter				■	■	■
Stipa capensis Thunb.		■	■			
Teucrium capitatum L.	■	■	■		■	
Thymus sibthorpii Benth.		■	■	■	■	■
Trifolium angustifolium L.					■	
Trifolium stellatum L.	■	■	■			
Verbascum leucophyllum Griseb.	■	■	■			
Verbascum undulatum Lam.			■		■	
**Number of species**	23	34	29	11	26	13
